# Enhanced Antioxidant and Anti-bacterial Potential of *Brugmansia suaveolens* Conjugated Chitosan Nanoparticles

**DOI:** 10.2174/0109298673417085251130062432

**Published:** 2026-01-13

**Authors:** Santhosh Kondajji Hanumanthappa, Anudeep Aldur Chandrashekar, Ravindranath Bilachi Sanganabassapa, Niranjana Pathappa, Goma Kathayat, Dinesh Rokaya, Manjunatha Hanumanthappa

**Affiliations:** 1 Department of Biotechnology and Bioinformatics, Kuvempu University, Jnanasahyadri, Shankaraghatta, 577451, Karnataka, India;; 2 Department of Biotechnology, Manipal Institute of Technology, Manipal Academy of Higher Education, Manipal, 576104, Karnataka, India;; 3 Department of Biochemistry, Kuvempu University, Jnana Sahyadri, Shankaraghatta, 577451, Karnataka, India;; 4 Department of Biochemistry, Manipal College of Medical Sciences and Teaching Hospital, Pokhara, Nepal;; 5 Clinical Sciences Department, College of Dentistry, Ajman University, Ajman, United Arab Emirates;; 6 Center of Medical and Bio-Allied Health Sciences Research, Ajman University, Ajman, United Arab Emirates;; 7 Department of Biochemistry, Jnana Bharathi Campus, Bangalore University, Bangalore, 560056, Karnataka, India

**Keywords:** Antioxidant, antibacterial, *Brugmansia suaveolens*, chitosan, encapsulation, nanoparticle

## Abstract

**Introduction:**

Chitosan nanoparticles (CNPs) are broadly explored for drug delivery due to their biocompatibility, biodegradability, and non-toxicity. This study encapsulated *Brugmansia suaveolens* leaf ethanol extract (BSLEE) into CNPs to enhance antioxidant and antibacterial activity.

**Materials and Methods:**

The optimization of synthesis, such as chitosan concentration of (1:1) to cross-link BSLEE, maintaining an optimal acidic pH (~4.5-5.5), and applying mild stirring at 50 rpm resulted in an encapsulation efficiency ranging from 26.33 to 48.58%, indicating the successful encapsulation of BSLEE within the CNPs. Characterization by UV-Vis, FTIR, SEM, XRD, EDX, and zeta potential confirmed successful formulation, with semi-crystalline, porous nanoparticles.

**Results:**

DPPH, FRAP, and total phenolics assay indicate BSLEE CNPs exhibited stronger antioxidant activity in the range of 30.42 ±0.77% to 55.85± 0.69% of DPPH inhibition, 34.73±2.71 to 121.44±1.83 µg/ml FSE, and 58±2.27 to 149.5±2.48 µg GAE/ml, respectively, when compared to crude BSLEE. The antibacterial effectiveness of BSLEE CNPs against *S. aureus* and *E. coli* was more significant when compared to the BSLEE and chitosan alone, attributed to enhanced membrane permeability and disruption of the bacterial cell membrane.

**Discussion:**

Antibacterial studies showed significant inhibition against *S. aureus* and *E. coli*, linked to improved membrane disruption. The BSLEE CNPs demonstrated promising biocompatibility and potential for application in antimicrobial and antioxidant therapies, warranting further clinical validation and *in vitro* findings of BSLEE-conjugated chitosan nanoparticles.

**Conclusion:**

The BSLEE CNPs showed promising pharmacological properties like antibacterial efficacy against *E. coli* and *S. aureus*, indicating their potential as an alternative approach to combat bacterial infections.

## 1 INTRODUCTION

Chitosan is a naturally occurring biocompatible polymer obtained from chitin and known as deacetylated chitin, found in a crystalline microfibril in nature. Chitosan exists in other polymorphic forms, such as α-chitin, and has a more crystalline structure, making it more stable and less soluble in water. β-chitosan is more soluble in water and has less crystallinity than α-chitosan, while γ-chitosan typically has an intermediate solubility and crystallinity. The efficiency of chitosan in a variety of claims directly depends on the degree of deacetylation [1, 2]. The chitosan solubility is based on various factors such as molecular weight, degree of acetylation, pH, temperature, and polymer crystallinity [3]. Since chitosan nanoparticles have unique properties like muco-adhesiveness, the major components of mucus, such as mucin, are negatively charged. This enables the electrostatic interaction that allows the nanoparticles to adhere to the mucosal surface, resulting in extended contact time [4]. As a result, drug retention is improved, leading to enhanced therapeutic effectiveness and enabling targeted and controlled drug delivery [5]. Chitosan is pH sensitive, which is soluble under acidic conditions due to protonation of amine groups and precipitates at neutral to basic pH [3]. Chitosan is actively used for its antioxidant activity and as a carrier molecule in drug delivery within the *in vivo* pharmacological field [6, 7]. In dentistry, chitosan nanoparticles are becoming a popular biopolymer for use in therapeutic interventions [8]. The chitosan has biocompatibility and anti-inflammatory effects, and holds the potential outcomes in wound healing and periodontal care following tooth extractions [9]. In addition, chitosan is biocompatible and has been shown to have a positive response for osteoconduction.

*Brugmansia suaveolens* (Bercht. & Presl., Solanaceae), popularly known as “Angel’s trumpet, Brazil's white angel trumpet, also known as angel's tears and snowy angel's trumpet”. *B. suaveolens* is cultivated in most of the houses of Chikmagalur (D) as an edge and horticultural plant. *B. suaveolens* contains many potent secondary metabolites, and various disease conditions like abscesses, pain, ulcers, and fungal infections of the skin were effectively treated using these compounds [10]. *B. suaveolens* leaves ethanol extract (BSLEE) can be prepared by a sequential cold extraction method [11]. Furthermore, it is used to treat and alleviate menstruation (dysmenorrhea) and serves as a vaginal antiseptic in white secretions. *B. suaveolens* typically contains tropane alkaloids, scopolamine (0.72-0.86 mg/g) and atropine (0.79-0.96 mg/g) were identified and quantified in the dry leaf of *B. suavelens* by GC-MS chromatogram [10]. These tropane alkaloids possess effective anticholinergic, antimicrobial, antispasmodic, and cytotoxic effects. Additionally, phenolic and flavonoid compounds present in *B. suavelens* are known for their antioxidant activity [12], and exhibit synergistic effects when encapsulated within chitosan nanoparticles. Both stimulating the central nervous system and simultaneously depressing the peripheral nerves, a characteristic typically of parasympathomimetics [13]. In our previous study, *B. suaveolens* leaf methanol extract conjugated silver nanoparticles also have potent anti-analgesic properties by targeting pain receptors when delivered through nanoparticles [14].

Many researchers have discovered that plant extracts encapsulated in chitosan nanoparticles are more potent in drug delivery. The valuable assets, like responsiveness to stimuli, muco-adhesion, surface modifiability, and biodegradability, make chitosan nanoparticles a better choice for drug delivery [15]. Hence, *B. suaveolens* encapsulated chitosan nanoparticles may change the absorption, distribution, metabolism, and excretion (ADMET) properties and enhance pharmacokinetics, bioavailability, lower toxicity, and therapeutic results. However, the antioxidant and antimicrobial properties were not investigated for BSLEE-encapsulated chitosan nanoparticles. Hence, in the present study, *B. suaveolens* leaves ethanol extract (BSLEE) was encapsulated within the chitosan nanoparticles (CNPs) to enhance the bioavailability and biocompatibility.

## 2 MATERIALS AND METHODS

### 2.1 Materials and Reagents

Young leaves of *B. suaveolens*, petroleum ether (Boiling point 40-60°C), and ethanol were purchased from Sisco Research Laboratories P. Ltd., India. Chitosan (200-600 mPa ֗S, 0.5% in 0.5% acetic acid at 200C) was purchased from Tokyo Chemical Industry Co. Ltd (Japan), Acetic acid glacial (catalog No.AS001-500 ML) and 2,2-diphenyl-1-picrylhydrazyl (DPPH, Mol.Wt:394.32), sodium hydroxide, Trichloro acetic acid (TCA), potassium ferricyanide and ferric chloride from HIMEDIA (Mumbai, India). Ferrous sulphate, folin-ciocalteu reagents, and gallic acid, phosphate-buffered saline (PBS) from Merck (Germany). Analytical grade ascorbic acid and sodium carbonate were purchased from Sigma-Aldrich (USA). Antimicrobial activity was examined by using *Escherichia coli* (*E. coli*, ATCC 8739) and *Staphylococcus aureus* (*S. aureus*, ATCC 6538) bacterial strains.

### 2.2 Plant Material Collection and Preparation of Plant Extract

The young leaves of *B. suaveolens* were collected during the flowering stage in 2024, near Aldur (p), Chikmagalur (d), Karnataka, India. Authentication was done by Prof. Y L Krishnamurthy, and the voucher specimen was deposited at the Department of Applied Botany, Kuvempu University, Shankaraghatta, Shimoga, Karnataka, India. Voucher No: KU/AB/HS/1010. The leaves were gently washed with distilled water and shade-dried at room temperature. The shade-dried leaves were pulverized into a coarse powder and stored in a sealed container for further use. The powdered leaf material (400 g) was subjected to sequential cold extraction by using Petroleum ether, ethanol, and water for 48h. interval of time. Further, the extract was concentrated using a rotary evaporator (RAPIDVAP Rotary Evaporator, Labconco. Corporation, USA) and kept at 4°C for further use.

### 2.3 Encapsulation of *B. suaveolens* with Chitosan

Encapsulation of *B. suaveolens* leaves ethanol extract (BSLEE) with chitosan nanoparticles (CNPs) was prepared as described by El-Naggar *et al.* 1% (*w/v*) concentration of Chitosan was dissolved in 1% acetic acid and stirred magnetically for 24 hours to ensure complete dissolution. Once dissolved, 1N sodium hydroxide was used to maintain the pH at 5. Chitosan solution and BSLEE solution (1:1) in equal volumes were mixed and incubated at room temperature. Following incubation, the resultant turbid mixture was centrifuged for 10 min at 10,000 × g, rinsed with distilled water, and subsequently freeze-dried for experimental and characterization purposes [16].

### 2.4 Determination of Encapsulation Efficiency

The BSLEE encapsulated CNPs encapsulation efficiency was determined by the method reported by Peighambardoust *et al.* [17], λmax was determined for several concentrations of BSLEE by utilizing a spectrophotometer at 295 nm (λmax). A standard curve was created based on readings of various concentrations, and a trend line was derived using linear regression with a standard equation (y = 0.0011x + 0.0106 and an R2 value (0.9962). After that, BSLEE-encapsulated CNPs were centrifuged at 16,000 g for 25 min. The obtained supernatant was measured by a spectrophotometer at 295 nm. The encapsulation efficiency of BSLEE-encapsulated CNPs was quantified using the following Eq. **1**:

Encapsulation efficiency = BSLEE encapsulated CNPs /Total BSLEE × 100…         (1)

### 2.5 Characterization of BSLEE Encapsulated CNPs

BSLEE encapsulated chitosan NPs were prepared and then subjected to characterization techniques using UV-visible spectroscopy, SEM, FTIR, EDX, XRD, and ZETA potential measurements. These techniques provide comprehensive insights into the nanoparticles' size, structure, behavior, particle distribution, functional group, elemental composition, and optical and surface properties.

#### 2.5.1 UV-visible Spectra of BSLEE CNPs

The peak absorbance wavelength of the BSLEE-encapsulated CNPs was determined by spectral analysis using a Shimadzu UV-1800 spectrophotometer (Japan) at varying wavelengths from 200 to 400 nm [16].

#### 2.5.2 Fourier Transform Infrared Spectroscopy (FTIR) Analysis

The FTIR spectra of freeze-dried BSLEE encapsulated CNPs were obtained by grinding them with KBr pellets, and subsequent analysis was conducted using an FTIR spectrometer (Bruker FTIR Alpha, Germany) over the spectral range spanning 4500-500 cm^-1^ with a resolution of 1 cm^-1^ [16].

#### 2.5.3 Scanning Electron Microscopy (SEM) Analysis

The surface morphology of the BSLEE-encapsulated CNPs was examined using a Hitachi SU-1500 (Japan) scanning electron microscope. The sample was dried on a glass slide and mounted over aluminum stubs; later, thin layers of gold were sputtered on the samples and analyzed with an electron microscope [17].

#### 2.5.4 X-ray Diffraction (XRD)

The X-ray diffraction analysis of the BSLEE CNPs was examined within a 2θ range from 10° to 60° using an X-ray diffractometer equipped with a copper (Cu) target (Bruker D2 Phaser second Gen using a Ni-filtered Cu Kα radiation (λ=1.54 A°), Germany). The instrument operated at 40 kV and 30 mA with a scan speed of 2° per minute [18].

#### 2.5.5 Energy Dispersive X-ray (EDX) Spectroscopy Analysis

The elemental composition for the BSLEE CNPs was investigated using (JSM-5800, JEOL) Energy Dispersive X-ray (EDX) spectroscopy [19].

#### 2.5.6 Zeta Potential of the BSEL-loaded CNPs

The BSLEE encapsulated CNPs’ surface charge, stability, and interaction with other molecules were studied through zeta potential analysis through the detection angle of 900 at 250 degrees using microtrac FLEX (Zetrac-W3232, version 11.0.0.2) [20].

### 2.6 Antioxidant Activity

#### 2.6.1 DPPH Scavenging Assay

DPPH (2, 2-diphenyl-1-picrylhydrazyl) free radical scavenging activity of BSLEE encapsulated CNPs was determined according to the method reported by Bagheri *et al.* [21]. 3ml of 0.12 mM ethanolic DPPH solution was added separately to 100, 300, and 500 µg/mL of chitosan, BSLEE encapsulated CNPs, and crude BSLEE, and the mixture was kept in the dark. The absorbance was recorded using a UV-Vis Spectrophotometer at 517 nm. A control sample, devoid of BSLEE-encapsulated CNPs, was prepared, and ethanol was employed as a baseline correction. The percentage of DPPH radical scavenging capacity was determined using the following equation.

Percentage (%) of DPPH radical scavenging capacity = [(A0− As) ∕A0] × 100

Where A0 and as represent the absorbance of the control and samples, respectively.

#### 2.6.2 Ferric Reducing Antioxidant Power (FRAP) Assay

Ferric reducing antioxidant power assay of BSLEE encapsulated CNPs was determined as described by Vifta *et al.* [22] with some modification: 1 mL of 0.2N phosphate buffer with pH maintained to 6.6 was separately added to 100,300, and 500 µg/ml of chitosan, BSLEE encapsulated CNPs, and crude BSLEE, and 1 mL of 1% potassium ferricyanide was added and incubated for 20 min. at 50°C. Following incubation, 1 mL of 10% TCA solution was added to the mixture. The resulting solution was subjected to centrifugation at 3000 rpm for 10 min. If a sediment was formed, the upper layer was collected, and subsequently 0.5 ml of ferric chloride and 10 ml of distilled water were added; finally, absorbance was recorded at 595 nm.

#### 2.6.3 Determination of Total Phenolic Contents (TPC)

The TPC of the BSLEE encapsulated CNPs was examined using the Folin-Ciocalteu (FC-reagent) method as described by Esmaeili *et al.* [23], 1mg/1ml of chitosan, BSLEE encapsulated CNPs, and crude BSLEE were prepared, and 100, 300, and 500 µg/ml of each sample were combined with 2.5 ml of 10% Folin-Ciocalteu reagent (*v/v*). After a 5 min incubation, 2 ml of 7.5% sodium carbonate (*w/v*) was added to the mixture. The mixture was then kept at room temperature for 2 h. Then the mixture was subjected to ultra-centrifugation for 3 min, and the resulting supernatant was collected. The absorbance of the supernatant was recorded at 765 nm using a spectrophotometer. Then the phenolic content in BSLEE encapsulated CNPs and crude BSLEE was expressed in µg Gallic acid equivalent per ml of BSLEE.

### 2.7 Antibacterial Property

The antibacterial activity of BSLEE-encapsulated CNPs, Crude BSLEE, and Chitosan was evaluated by using the disc diffusion method [24], with some modification using Muller-Hinton agar (MHA) medium. Actively growing (overnight grown) culture of *Escherichia coli* (ATCC 8739) and *Staphylococcus aureus* (ATCC 6538), 10 µl was uniformly spread on Petri plates containing the medium using an L-shaped glass rod. Sterile filter disc (6 mm diameter) impregnated with BSLEE encapsulated CNPs and chitosan alone dissolved in 1% acetic acid, and crude BSLEE dissolved in 99.9% ethanol at various concentrations, along with standard ampicillin (20 µg/ml). Then petri plates were incubated at 37°C for 24 h.

## 3 RESULTS

In the present study, CNPs were synthesized from leaves of ethanol extract of *B. suaveolens,* and their morphological, physiological, antioxidant, and antibacterial activity was evaluated.

### 3.1 *Brugmansia Suaveolens* Leaves Ethanol Extraction

In this study, about 400g of *B. suaveolens* leaves material is subjected to cold extraction using petroleum ether, ethanol, and aqueous solvents in order to obtain the crude extract. The amount of yield obtained was 8.899 g, 10.096 g, and 7.754 g, respectively.

Various methods have been used for the synthesis of BSLEE CNPs. *B. suaveolens* was incorporated into the chitosan matrix during nanoparticle synthesis while considering the stability, particle size, and safety of CNPs. *B. suaveolens* contains many biomolecules. Biological molecules can act as stabilizing or reducing agents during nanoparticle synthesis [25]. After the BSLEE CNPs solution was prepared, it was freeze-dried to obtain a powder form with a porous texture.

### 3.2 Characterization of BSLEE CNPs

#### 3.2.1 UV-Visible Spectra of BSLEE CNPs

UV-visible spectrophotometric analysis of the synthesized BSLEE CNPs revealed a maximum absorption band in the range of 200-400 nm. This generally corresponds to the Band II region, typically associated with the benzoyl radical (A ring) of the flavonoid structure, which usually appears between 240-280 nm and is indicative of the presence of carbonyl (C=O) groups [26]. The occurrence of carboxylic or carboxyl groups may result from the degradation of chitosan, forming new side groups in the degraded polymer chain [27]. The observed absorption peak suggests a π−π* electronic transition within the BSLEE CNPs, reflecting the molecular structure and degree of encapsulation of BSLEE [28].

*Brugmansia suaveolens* is known to contain secondary metabolites such as tropane alkaloids like scopolamine, atropine, and hyoscyamine [29, 30]. Scopolamine exhibits a characteristic UV absorption at 257 nm, while atropine absorbs near 258 nm. Although these standard values are slightly lower than the 265.5 nm peak shown in Fig. (**1A**), they may contribute to the overall absorbance or reflect a broader absorption band in this region. Another tropane alkaloid, hyoscyamine, also absorbs at 248, 255, 260, and 265 nm with slight shifts depending on the solvent used for solubilization [31].

#### 3.2.2 Fourier Transform Infrared (FT-IR) Spectroscopy Analysis

FT-IR analysis was employed to characterize and identify the functional groups present within synthesized BSLEE CNPs. The FT-IR spectrum of BSLEE CNPs reveals significant absorption bands in the range of 561 cm^-1^ to 2926 cm^-1^ (Fig. **1B**). A significant band at 2926 cm^-1^ signifies the asymmetric stretching vibration of the methylene (CH_2_) group, which indicates the occurrence of the methylene group within the chitosan backbone [32]. The absorption at 1620 cm^-1^ corresponds to C=O groups, due to the presence of carboxylate groups (COO^-^ band), which exhibit absorption bands at lower wave numbers [33]. The band observed at 1542 cm^-1^ corresponds to the occurrence of NH_2_ and NH deformations in amides [34]. The observed peak at 138 2cm^-1^ is associated with the symmetric bending vibration of a methyl group (CH_3_) within the N-acetyl (NHCOCH_3_) functional group [35]. The peak at 1056 cm^-1^ corresponds to C-N bending and stretching, which indicates the formation of a complex between chitosan and BSLEE [36]. The absorption peak at 1015 cm^-1^ corresponds to the C-O-C bending vibration and C-OH stretching vibration [37]. The absorption peak at 639 cm^-1^ is associated with the hydrophobicity of the BSLEE and chitosan composite [38]. Finally, the absorption band at the 561 cm^-1^ region corresponds to the C-Br medium stretching of the alkyl halide group [39].

#### 3.2.3 Energy-dispersive X-ray Spectroscopy (EDX) Analysis

The chemical composition and major elements of BSLEE CNPs were analyzed using Energy-dispersive X-ray spectroscopy. Fig. (**1C**) shows EDX analysis reveals the presence of carbon, nitrogen, oxygen, chlorine, and potassium. The EDX predicts carbon (C) presence the characteristic X-ray peak at 0.28keV with a weight of 35.75%, nitrogen (N) exhibits X-ray peak around 0.39keV with 14.44% of weight, and oxygen (O) produces a high characteristic X-ray peak around 0.5 to 0.6keV with a weight of 48.10%. chlorine (Cl) and potassium (K) are the elements that show X-ray peaks in the energy range around 2.6 to 2.8 keV and 3.0 to 3.5 keV with 1.41 and 0.29% of weight, respectively.

#### 3.2.4 Zeta Potential of the BSLEE-encapsulated CNPs

The zeta potential of BSLEE-encapsulated chitosan nanoparticles (CNPs) is a critical parameter for understanding their colloidal stability and interaction behavior. Specifically, the surface charge of chitosan plays a key role in determining how the nanoparticles behave in suspension, how they interact with negatively charged biomolecules, and how effective they are in various applications such as drug delivery or environmental remediation. This zeta potential is influenced by several factors, including the pH of the medium, the ionic strength of the surrounding solution, and the presence of other interacting molecules or ions. Fig. (**1D**) illustrates the size distribution, zeta potential, and polydispersity index (PDI) of the BSLEE-encapsulated CNPs, as measured using a laser diffractometer under a field strength of 5 kV/m. The nanoparticles exhibited a wide size range, from 321 to 2729 nm. When chitosan is dissolved in acetic acid, its amino groups are protonated to form NH_3_^+^, imparting a positive charge to the nanoparticles [40]. However, at pH 5, the measured zeta potential was only +2.5 mV, indicating a very low surface charge. This reduced positive charge is insufficient to maintain strong electrostatic repulsion between particles, which compromises suspension stability and weakens interactions with negatively charged species [41]. Therefore, the relatively low zeta potential at pH 5 suggests limited electrostatic stabilization and reduced functional performance of the nanoparticles under these conditions.

#### 3.2.5 Scanning Electron Microscopy (SEM) Analysis

A bio-synthesized chitosan nanoparticle prepared from BSLEE is depicted in Figs. (**2A**-**C**). As a result, BSLEE may reduce chitosan molecules and the related chitosan nanoparticles in a way that is cost-effective, safe, and environmentally beneficial. This was supported by our SEM analyses of the BSLEE-encapsulated chitosan nanoparticles, which clearly showed highly porous scaffold-like microstructures. The porous nature of the scaffolds provides increased internal space and surface area, thereby enhancing the loading capacity of bioactive compounds and improving encapsulation efficiency. Additionally, the scaffold's porosity, pore size, distribution, and interconnectivity significantly influence cell proliferation, tissue regeneration, and distribution of nutrients and oxygen, thereby enabling diffusion-controlled release kinetics of BSLEE [42, 43]. In addition, in the nanoparticle’s bio-fabrication process, secondary metabolites from ethanolic extracts of plants were employed as reducing agents and may play the functions of stabilizers, reducers, or maybe both during the encapsulation of nanoparticles. Increased distribution of scaffold-like microstructures, enhanced treatment, stability, absorption, and the possibility to encapsulate more pharmacological components are all made possible by smaller particle sizes.

#### 3.2.6 X-ray Diffraction (XRD)

A wide variety of techniques have been used in the synthesis of BSLEE-encapsulated chitosan nanoparticles. Both the stability and safety of the BSLEE CNPs, as well as their particle size, should be considered when selecting an acceptable technique for their production. To determine the crystallinity of the synthesized chitosan nanoparticles, an XRD examination was carried out. To do this, the XRD diffraction patterns were recorded, as shown in Fig. (**2D**). The results showed the existence of many distinctive peaks, indicating that the chitosan nanoparticles are semi-crystalline in nature. The semi-crystalline polymer has revealed a state of crystalline and amorphous nature; the crystalline areas are densely packed, providing rigidity, mechanical strength, and controlling drug diffusion due to being less permeable, while the amorphous regions provide flexibility, elasticity, and a more porous nature, facilitating quicker drug diffusion. The semi-crystalline property of BSLEE CNPs provides a tendency to withstand stress and physiological conditions, especially in biomedical applications, facilitating effective drug delivery to target tissues [44, 45].

The peaks are located at angles (2θ) 10.10°, 21.9°, 37.10°, and 44.45°, indicating the direction of the chitosan semi-crystalline nanoparticle. Because of its semi-crystalline nature, chitosan exhibits a broad peak that appears at 2θ within the range of (15-25º). The strongest and most prominent peak in the chitosan's XRD pattern was found at 44.45°, indicating that the material had a high degree of crystallinity. A prominent diffraction peak at 2θ=20-25° can be seen in the spectra; this was determined to be the main diffraction peak of (110 nm) chitosan. The diffraction peaks at 2θ of 10.10° and 21.9° were seen in the XRD diffraction patterns of the chitosan nanoparticles are semi-crystalline in nature. Every one of these diffraction peaks reflects the anhydrous α-chitin's (110 nm) and (020 nm) hydrated semi-crystalline structure. This suggests that the synthesized chitosan nanoparticles have a semi-crystalline state.

### 3.3 Encapsulation efficiency of BSLEE CNPs

Based on the standard curve, which is obtained from the crude BSLEE with a standard equation (y = 0.0011x + 0.0106 and an R2 value (0.9962) as shown in Fig. (**3A**), the encapsulation efficiency of BSLEE CNPs was found in between 26.33 ±0.45% to 48.58 ±0.93. A chitosan/BSLEE ratio of 1:0.25 shows the highest amount of BSLEE (48.58%). The different ratios of chitosan/BSLEE like 1:0.50, 1:0.75, and 1:1 exhibit 36.56 ±0.68, 31.33 ±0.45, and 26.33 ±0.45% encapsulation efficiency, respectively (Table **1**). In this study, as the amount of BSLEE in CNPs increases, the encapsulation efficiency of BSLEE decreases. The primary reason for this decreasing trend is due to the saturation level of BSLEE, and excess BSLEE cannot be absorbed by the chitosan matrix [46]. Other factors, like the high molecular weight of the molecules, typically restrict the movement of the sample and result in lower encapsulation efficiency [47].

### 3.4 Antioxidant Activity

#### 3.4.1 DPPH Radical Scavenging Assay

The DPPH radical scavenging assay was employed to evaluate the free radical scavenging activity of BSLEE CNPs when compared to its crude BSLEE. BSLEE CNPs have a substantially stronger antioxidant potential activity in the range of 30.42 ±0.77% to 55.85± 0.69%, which is considerably more than that of crude BSLEE in the range of 22.25±0.60% to 48.65±0.51%. Additionally, chitosan showed minimal scavenging activity in the range of 1.03±0.25% to 3.96±0.77% antioxidant activity (Fig. **3B**). Furthermore, increasing the BSLEE CNPs enhances the antioxidant activity, which may be attributed to the shielding effect of encapsulation, which declines the evaporation rate through the controlled release of BSLEE [48].

The DPPH scavenging activity of BSLEE CNPs was higher than crude BSLEE, primarily due to free amino groups present in chitosan, which donate hydrogen atoms to neutralize DPPH radicals [49, 50]. Additionally, low molecular weight chitosan derivatives exhibit a higher radical scavenging capacity [50]. Therefore, both the high encapsulation efficiency and synergistic interactions between chitosan and BSLEE contribute to the enhanced DPPH scavenging activity. This dual effect highlights the value of chitosan as an effective nanocarrier in antioxidant delivery systems.

#### 3.4.2 Ferric Reducing Antioxidant Power (FRAP) Assay

The ferric-reducing antioxidant capacity of BSLEE CNPs, crude BSLEE, and chitosan was evaluated using the FRAP assay. FRAP assay assesses the reduction of ferric ions (Fe^3+^) to ferrous ions (Fe^2+^), with higher absorbance values at 595 nm, indicating greater reducing power and thus higher antioxidant capacity. The antioxidant capacity (µg/ml FSE) of BSLEE CNPs obtained in the range of 34.73±2.71 to 121.44±1.83µg/ml FSE, for crude BSLEE antioxidant obtained in the range of 26.75±1.16 to 104.59±1.57µg/ml FSE, and chitosan, which exhibits minimal antioxidant capacity in the range of 1.91±0.91 to 7.465±1.18µg/ml FSE (Fig. **3C**).

A strong correlation was observed between FRAP and TPC because, FRAP assay is a method used to assess the electron-donating capacity of antioxidants present in BSLEE CNPs. Chitosan alone has mild reducing capacity; hence, encapsulations also enhance controlled release of phenolics and enhance antioxidant performance over time, as observed in FRAP assays [51]. Phenolic compounds are excellent electron donors due to their hydroxyl groups. Therefore, higher total phenolic content (TPC) typically correlates with increased FRAP values, indicating stronger antioxidant activity [52].

#### 3.4.3 Total Phenolic Content (TPC)

The total phenolic content of BSLEE CNPs, crude BSLEE, and chitosan was quantified using Folin-Ciocalteu (FC-reagent) with a standard curve equation y=0.0031x+0.0241, with an R2 value of 0.9978, which was employed to calculate the total phenolic content from the absorbance (OD) value at 765 nm. Total phenolic content for BSLEE CNPs obtained in the range of 58±2.27 to 149.5±2.48µg GAE/ml, crude BSLEE exhibits TPC in the range of 25.63±2.48 to 101.26±3.19µg GAE/ml, and chitosan has negligible TPC (Fig. **3D**). The phenolic compounds are the main reason for antioxidant activity due to their redox properties [53].

### 3.5 Antibacterial Activity of BSLEE Encapsulated CNPs

The antibacterial activity of BSLEE encapsulated CNPs, crude BSLEE, and chitosan alone was assessed against *S. aureus* and *E. coli* using the disc diffusion method. BSLEE encapsulated CNPs at a concentration of 100 µg/ml exhibited inhibition zones of 0.89±0.01 cm and 0.81±0.02 cm against *S. aureus* and *E. coli*, respectively. As the concentration increased to 200 µg/ml, the inhibition zones expanded to 1.14±0.02 cm for *S. aureus* and 1.03±0.04 cm for *E. coli*. The maximum antibacterial activity was observed at 300 µg/ml, with inhibition zones of 1.37±0.04 cm for *S. aureus* and 1.23±0.03 cm for *E. coli*. The positive control, ampicillin at a concentration of 20 µg/ml shows 1.46±0.03cm and 1.30±0.01 cm zone of inhibition for *S. aureus* and *E. coli,* respectively. The crude BSLEE at the concentrations 100, 200, and 300 µg/ml does not show any zone of inhibition for either of the bacteria. As the concentration increased to 1200 µg/ml, a 0.78±0.03 cm zone of inhibition was observed only for *S. aureus.* Further increasing the concentration to 2400 µg/ml exhibited 1.2±0.01 cm and 0.8±0.14 cm zone of inhibition for both bacteria (Figs. **4A**-**F**). Positive control ampicillin shows a similar trend as the BSLEE encapsulated CNPs sample with 1.45±0.02 cm and 1.08±0.04 cm for *S. aureus* and *E. coli,* respectively. Negative control, 1% acetic acid for BSLEE CNPs and 99.9% ethanol for crude BSLEE, does not show any zone of inhibition for either of the bacteria. Furthermore, chitosan alone exhibited 0.55±0.03 cm and 0.59±0.01 cm zones of inhibition against *S. aureus* and *E. coli,* respectively (Table **2**).

## 4 DISCUSSION

In the present study, a comprehensive investigation was conducted to assess the potential of *B. suaveolens* encapsulated chitosan nanoparticles (BSLEE CNPs). The study includes detailed characterization, antioxidant (DPPH, FRAP, TPC), and antibacterial potential against *E. coli* and *S. aureus*. The findings revealed that BSLEE CNPs exhibited improved functional characteristics, including enhanced antioxidant activity and notable antibacterial efficacy compared to crude BSLEE. Various methods have been used to synthesize the chitosan CNPs, including adding a cross-linking agent, sodium tripolyphosphate (TPP). However, we directly synthesized BSLEE CNPs by single-step bio-reduction, which is eco-friendly and non-toxic, as described [54].

Characterization of synthesized BSLEE CNPs was primarily employed through UV-visible spectrophotometric analysis, which confirmed the formation of BSLEE CNPs. The maximum absorbance wavelength at 230 nm indicates bio-fabrication of BSLEE CNPs. The BSLEE acts as a reducing agent that might be bound to cationic NH_2_ groups of chitosan, which is indicated in the FT-IR absorption spectrum at 1542 cm^-1^ corresponding to the occurrence of NH_2_ and NH deformations in amides. According to Ali *et al.* [36] The peak at 1056 cm^-1^ corresponds to C-N bending and stretching, which specifies the foundation of a complex between chitosan and BSLEE. The occurrence of the strongest peak of carbon (C) and oxygen (O) in the energy-dispersive X-ray analysis confirms that the BSLEE was successfully coated on the chitosan backbone [55] along with the presence of nitrogen, chlorine, and potassium. Zeta potential analysis predicted that the observed size of BSLEE CNPs obtained in the range of 321 to 2729 nm and a zeta potential value of 2.5 mV at pH 5, as reported by Shetta *et al.* 2019 [56]. Zeta potential values around 5 mV align with fast aggregation of CNPs and low stability. Furthermore, SEM analysis exhibited highly porous scaffold-like microstructures (Figs. **2A**-**C**), indicating higher surface area and biocompatibility. The XRD analysis confirms the semi-crystalline nature of BSLEE CNPs. The presence of diffraction peaks is located at angles (2θ) 10.10°, 21.9°, 37.10°, and 44.45°, aligning with the semi-crystalline state and with the prominent peak at 44.45° representing a high degree of crystallinity.

The encapsulation efficiency of BSLEE CNPs exhibits a similar trend as the amount of BSLEE in CNPs increases; the encapsulation efficiency of BSLEE decreases. Our results were closely aligned with findings reported by Soltanzadeh *et al.* [57], for pomegranate peel extract within the chitosan NPs, encapsulation efficiency was obtained in the range of 26.3-69.7% and Hasani *et al.* [58] reported encapsulation efficiency for lemon essential oil in chitosan-Hicap nano gels obtained in the range of 68.09±0.71 to 85.43±2.35%. Various studies suggest that chitosan hinders the reactive oxygen species (ROS) and barricades lipid oxidation in food and biological systems [59]. The BSLEE CNPs exhibited significant antioxidant activity compared to crude BSLEE and chitosan. In the present study, BSLEE encapsulated within chitosan exhibits a higher antioxidant capacity due to the effective delivery system from encapsulation [60]. The BSLEE encapsulated within the chitosan alone exhibits antioxidant activity, which is directly attributed to increased surface area and smaller particle size, which improves the binding ability to the target [61]. Chitosan exhibits a minimum antioxidant activity of less than 10%, which is due to the free radicals reacting with the amino and hydroxyl groups present in the chitosan backbone [47]. A similar result was reported by Bagheri *et al.* [21], in which DPPH radical scavenging activity was in the range of 13.5-56.8% and 14.2-67.4%, for CNPs loaded with nettle essential oil and pure nettle essential oil, respectively. The key source for antioxidant, antiviral, antibacterial, and anti-inflammatory properties is due to the presence of several compounds such as cyanine, flavonoids, flavones, flavonols, and phenols [62, 63]. Variation in the antioxidant activity due to the amount or quantity, and diversity of polyphenolic compounds and phenolic hydroxyl groups in the plant [64]. The above antioxidant results indicate that BSLEE has reasonably lesser antioxidant activity when compared with BSLEE-encapsulated chitosan, which has more significant antioxidant properties.

Furthermore, the antibacterial efficacy of BSLEE CNPs, crude BSLEE, and chitosan was evaluated against *S. aureus* and *E. coli*. BSLEE CNPs exhibited notable antibacterial activity for both bacteria, whereas crude BSLEE required a higher concentration to exhibit antibacterial activity. The improved permeability of bacterial cell membranes and depolarization of chitosan effectively contribute to antibacterial activity [65]. According to Kheiri *et al.* and Rajesh Kumar *et al.* [66, 67] The highly positive surface nature of CNPs interacts with negatively charged biological membranes of tested pathogens, later altering the membrane permeability as a result, inhibiting the growth of the pathogens. Moreover, the antibacterial effects of chitosan depends on various types of microorganisms. The variation in gram-positive and gram-negative bacteria results in unlike sensitivity actions of chitosan. For instance, highly negatively charged cell surface gram-negative bacteria facilitate more binding affinity towards the phospholipid membrane of cationic chitosan at lower pH [68, 69]. The antibacterial efficacy of BSLEE-CNPs in direct comparison with other positively charged nanoparticles has not been explored. Hence, no such experimentally validated data are available in the existing literature. In the present study, our findings were consistent with the previous study [70], which reported summer savory (*Satureja hortensis*) EO-loaded chitosan NP antibacterial activity.

An *in vivo* acute toxicity study was carried out using a Wistar albino rat model to evaluate the safety of *Brugmansia suaveolens* leaf extract. A beneficial dose of 500 mg/kg b. w. was selected, calculated as 1/10^th^ of the LD_50_, for subsequent investigation in wound healing applications [71]. The cytotoxicity assay revealed that BSLEE exhibited lower toxicity in the L929 fibroblast cell line, with cell viability decreasing from 85.93% at 50 µg to 30.21% at 250 µg. The IC_50_ was determined to be 187.48 µg/ml. We didn’t examine the encapsulated chitosan nanoparticles. Chitosan nanoparticles in liver cells (HepG2 and WRL68) show dose-dependent minimal cytotoxicity at concentrations ≤0.5 mg/mL [72].

Finally, the finding of this study demonstrates that BSLEE CNPs exhibit significant antioxidant and antibacterial activities compared to crude BSLEE. Future research focuses on evaluating *in vivo* studies of BSLEE CNPs’ pharmacokinetics and pharmacodynamic response in Wistar rats or BALB/c mice models, particularly in the context of antimicrobial load reduction and survival rate, post-treatment with BSLEE CNPs in Methicillin-resistant *Staphylococcus aureus* (MRSA) or *E. coli* intraperitoneal infection, and oxidative stress-related conditions.

## CONCLUSION

This study highlights the potential of BSLEE as an effective antioxidant and antibacterial agent. The *in vitro* analysis demonstrated that the nanoparticles exhobbits significant scavenging activity, suggesting robust antioxidant properties due to secondary metabolites such as phenolic and polyphenolic compounds, for example chlorogenic acid, flavonoids such as kaempferol, alkaloids like scopolamine and atropine, and tannins. Additionally, they showed promising pharmacological properties like antibacterial efficacy against *E. coli* and *S. aureus*, indicating their potential as an alternative approach to combat bacterial infec-tions. Further *in vivo* studies and toxicological assessments are recommended to validate these *in vitro* findings of BSLEE-encapsulated CNPs.

## Figures and Tables

**Fig. (1):**
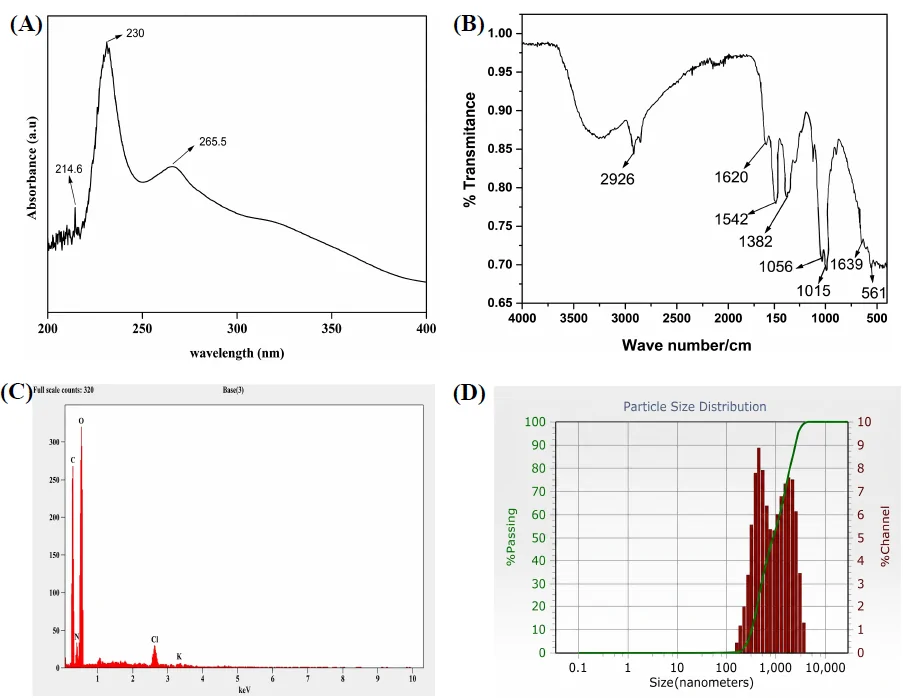
(**A**) UV-visible Spectra of BSLEE encapsulated CNPs, (**B**) FT-IR spectrum of BSLEE encapsulated CNPs, (**C**) EDX analysis graph for BSLEE encapsulated CNPs, (**D**) Zeta potential measure and size range of BSLEE encapsulated CNPs.

**Fig. (2):**
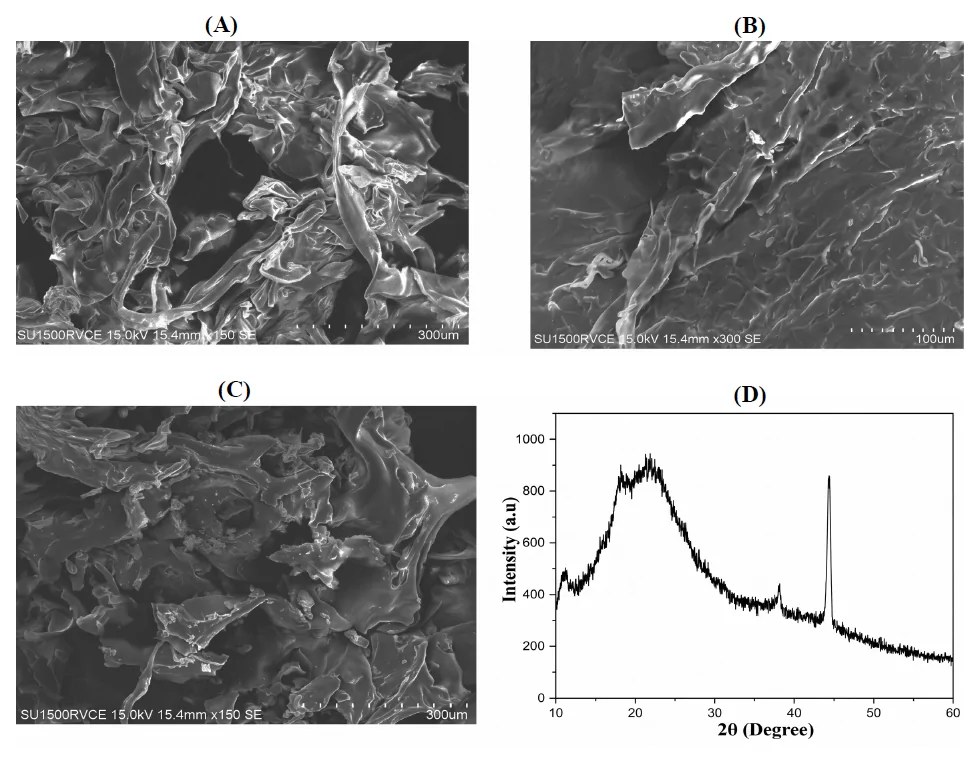
(**A-C**) Scanning electron microscopy micrographs of porous chitosan scaffolds, (**D)** X-ray diffraction pattern of semi-crystalline BSLEE encapsulated CNPs.

**Fig. (3):**
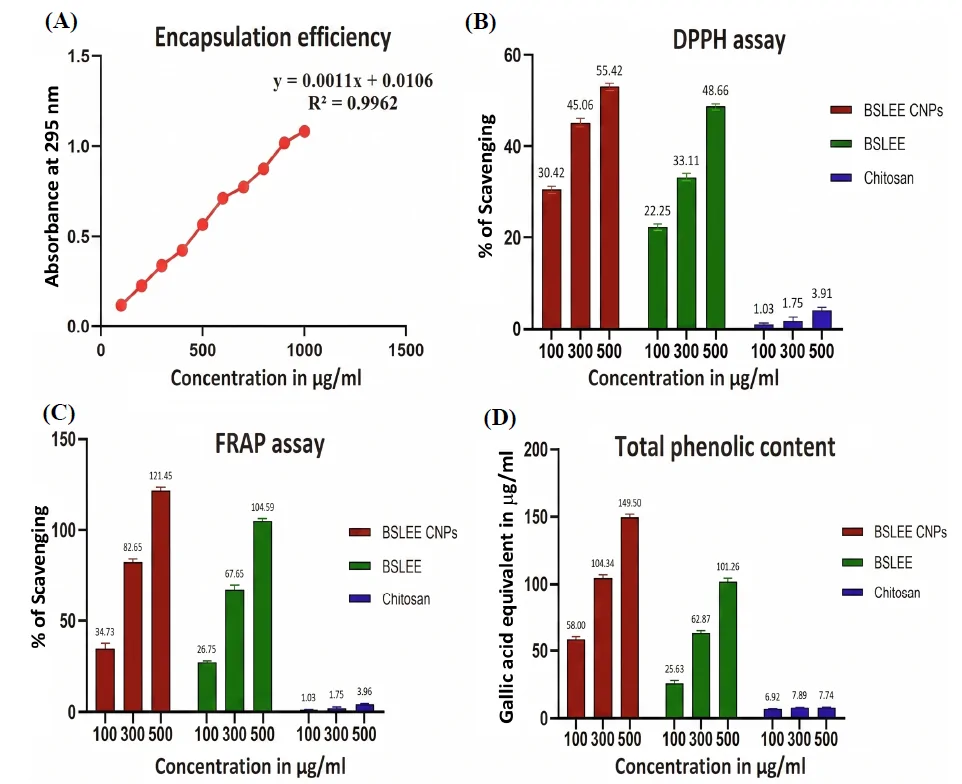
Antioxidant assay. (**A**) Standard curve of crude BSLEE for encapsulation efficiency, (**B**) DPPH radical scavenging activity, (**C**) FRAP Assay for BSLEE encapsulated CNPs, crude BSLEE and chitosan, (**D**) The total phenolic content (TPC) of BSLEE and chitosan.

**Fig. (4):**
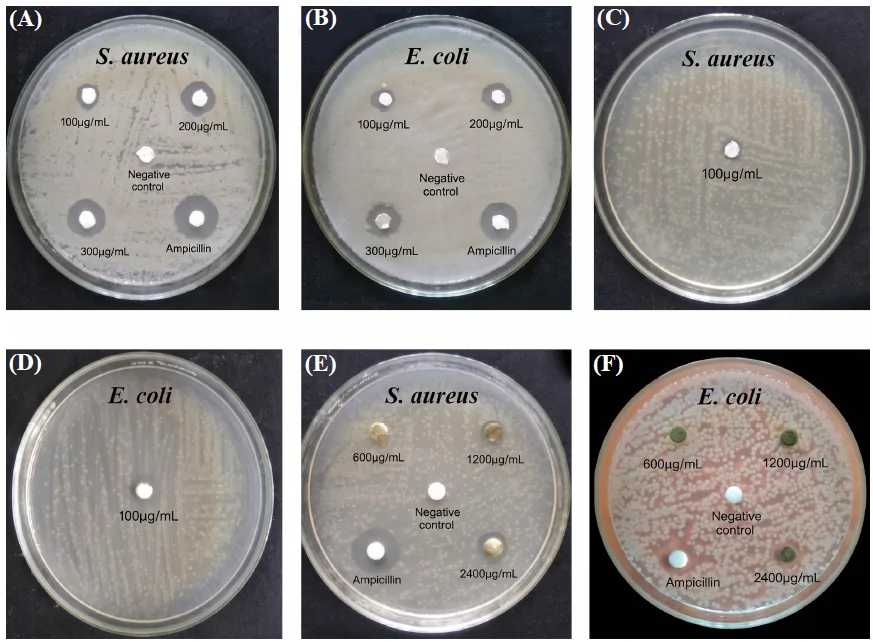
(**A**) Antibacterial activity of BSLEE encapsulated CNPs against *S. aureus,* (**B**) Antibacterial activity of BSLEE encapsulated CNPs against *E. coli,* (**C** and **D**). Antibacterial activity of chitosan alone against *S. aureus* and *E. coli,* respectively, (**E**) Antibacterial activity of crude BSLEE against *S. aureus*, (**F**) Antibacterial activity of crude BSLEE against *E. coli.*

**Table 1:** Encapsulation efficiency of BSLEE encapsulated CNPs determined by UV-Vis spectrophotometry.

**Encapsulation efficiency (Chitosan/BSLEE) (*W/W*)**	**Encapsulation percentage (%)**
1:0	0
1:0.25	48.58 ±0.93
1:0.50	36.56 ±0.68
1:0.75	31.33 ±0.45
1:1	26.33 ±0.45

**Note:** Each value is the mean of triplicate (± standard deviation).

**Table 2:** Antibacterial activity of BSLEE encapsulated CNPs and BSLEE.

**Test Sample**	**Concentration in μg/ml**	***S. aureus***	***E. coli***
BSLEE		**Zone of Inhibition in cm**	
	600	0.0	0.0
	1200	0.8	0.0
	2400	1.2	0.9
Standard Ampicillin	20	1.15	1.10
BSLEE CNPs	100	0.9	0.82
	200	1.15	1.05
	300	1.34	1.25
Standard Ampicillin	20	1.48	1.31

**Note:** Antibacterial activity of BSLEE encapsulated CNPs and crude BSLEE. Each value is the mean of duplicates (± standard deviation).

## Data Availability

The data presented in this study are available on request from the corresponding authors.

## LIST OF ABBREVIATIONS

BSLEE: *Brugmansia Suaveolens* Leaf Ethanol Extract
CNP’s: Chitosan Nano Particles
BSLEE CNPs: *Brugmansia Suaveolens* Leaf Ethanol Extract Encapsulated Chitosan Nanoparticles
ADMET: Absorption, Distribution, Metabolism, and Excretion
EDX: Energy-dispersive X-ray Spectroscopy
FT-IR: Fourier Transform Infrared
SEM: Scanning Electron Microscopy
XRD: X Ray Diffraction
DPPH: 2, 2-diphenyl-1-picrylhydrazyl
FRAP: Ferric Reducing Antioxidant Power
TPC: Total Phenolic Contents
